# The study of formulated Zoush ointment against wound infection and gene expression of virulence factors *Pseudomonas aeruginosa*

**DOI:** 10.1186/s12906-018-2251-4

**Published:** 2018-06-15

**Authors:** Maryam Meskini, Davoud Esmaeili

**Affiliations:** Department of Microbiology and Applied Microbiology Research Center, Systems Biology and Poisonings Institute and Department Of Microbiology, Baqiyatallah University of Medical Sciences, Tehran, Iran

**Keywords:** Pseudomonas aeruginosa, Burns, Satureja khuzestaniea, Zataria multiflora, Mentha Mozaffariani Jamzad, Honey, Polyurethane, Real-time RT-PCR, ZOUSH

## Abstract

**Background:**

The outbreak of MDR and XDR strains of *Pseudomonas aeruginosa* and increased resistance to infection in burn patients recommend the issue of infection control. In this research, we study ZOUSH herbal ointment for gene silencing of *Pseudomonas aeruginosa*.

**Methods:**

The herbal ZOUSH ointment was formulated by alcoholic extracts of plants *Satureja khuzestaniea*, *Zataria multiflora*, *Mentha Mozaffariani Jamzad*, honey, and polyurethane. The MIC and disk diffusion tests were examined by single, binary, tertiary and five compounds.

Three-week-old mice were considered to be second-degree infections by *Pseudomonas aeruginosa*. During the interval of 5 days, cultures were done from the liver, blood, and wound by four consecutive quarters and counting of *Pseudomonas aeruginosa* was reported in the liver.

In this study, silver sulfadiazine ointments and Akbar were used as a positive control. The gene gyrA reference was used as the control. Real-time RT-PCR results were evaluated based on Livak as the comparative Ct method.

**Results:**

The In vitro results indicated that wound infection was improved by healing wound size in the treatment groups compared to control treatment group. In this research, the changes in gene expression were evaluated by molecular technique Real-time RT-PCR. The results showed downregulation *exoS*, *lasA*, and *lasB* after treatment with ZOUSH ointment. SPSS Analyses showed that reduction of expressions in genes *exoS*, *lasA* and *lasB* after treatment with ZOUSH ointment were significantly meaningful (*p* < 0.05).

**Conclusion:**

Our study showed that ZOUSH ointment has the positive effect for gene silencing *Pseudomonas aeruginosa* in the mouse model with the second-degree burn. The positive effects decreased in the number of bacteria by reducing the expression of virulence bacteria genes as exoS, lasA and lasB and improvement of wound healing.

## Background

*Pseudomonas aeruginosa is* an opportunistic gram-negative bacil. When it is entered into a host cell, its employee several virulence factors such as cell-associated and extracellular factors [1].

This opportunistic bacterium is responsible for severe and fatal infections in patients with underlying diseases, such as Cystic fibrosis, chronic respiratory infections, Bronchiectasis, Severe Burns, Neoplasia, Neutropenia, Diabetes, AIDS and other diseases [2–4].

This bacterium is a common cause of nosocomial and burn wound infections [5]. Over than 75% of death after burn is related to *Pseudomonas* infection [6]. Each year, nearly 2.4 million burn wounds occur in all over the world that 650,000 patients needed to treatment and 75,000 cases are admitted to the hospitals, that the burning rate in 20,000 of these patients is above 25% [7]. In the year 2000, the burn has been responsible for the death of 238,000 patients in the whole world. In the year 2005, 2576 people in Iran and 406 people in Tehran province had died due to burn wounds [8].

According to conducted research, the mortality rate reported 30–50% for *Candida albicans*, 20–30% for *P. aeruginosa* and 5% for *Staphylococcus aureus* [9].

One of the most important side effects of burns is the infection which is caused by burn and war wounds. Dau and et al. reported that 7% of war wounds were related by burn wounds [10]. On the other hand, many researchers are studying and testing various groups of plants on the antibacterial properties of medicine plants.

Some plants such as *Zataria multiflora* and *Satureja khuzestanica* not only have many applications in traditional medicine but also they have phenolic components like thymol and carvacrol which have antimicrobial activities against fungi [11, 12].

Due to the resistance of *P. aeruginosa* to many antibiotics, the treatment should be selected based on the antibiotic susceptibility of the strain because resistant strains can quickly emerge during treatment. In addition to, certain clinical infections caused by *P. aeruginosa* should not be treated with one drug because the success rate of such treatments is low, and the possibility of developing drug resistance increase [13].

The presence of the efflux pump systems and β-lactamases contribute to the intrinsic resistance of *P. aeruginosa* to broad-spectrum penicillins, some cephalosporins, carbapenems, monobactams, fluoroquinolones, aminoglycosides, and colistins [14]. The possibility of acquired resistance to these antibiotics is rising due to mutations and the horizontal gene transfer through plasmids, integrons, and transposons. Studies have shown that *P.aeruginosa* isolates showed multiple-antibiotic resistance with rate 50% in Europe and about 35% in the United States [15, 16].

Iran with 11 different climates and more than 7500 plant species is regarded an excellent region to achieve valuable and rare medicinal species. Currently, 25% of the existing drugs are made from herbal sources, and 12% of the drugs are made from microbial sources [13].

Thyme, dried flowering plants, and *Zataria multiflora Boiss* are belonging to the family Labiatae with at least 6% of essential oil (volume/weight) [17–19].

Oregano (*Origanum vulgare L.*) from the family Lamiaceae has at least 0.1% (volume/weight) essential oil [20]. *Satureja khuzestanica* is an herbaceous, aromatic and perennial herb of the family Lamiaceae with numerous branches. *Satureja* is one of the genera of the family Lamiaceae belonging to the subfamily Nepetoideae and the tribe Mentheae [21, 22].

## Methods

### Preparation of alcoholic extracts

Alcoholic extracts of *S. khuzestanica*, *Z. multiflora*, and *O. volgarum* were prepared from Barij Essence Pharmaceutical Company in powder form. The compounds of the extracts were measured by gas chromatography (GC-MS) in Kashan Barij Essen [23].

### Preparation of ZOUSH ointment

The extracts were dissolved in 1/3 dimethyl sulfoxide, Honey was dissolved in about 1/3 of distilled sterile water and The Polyurethane was first dissolved in 6 M urea at a rate of 1/3 and then placed on indirect heat for evaporation of urea. This solution was prepared as a stock. The ZOUSH ointment is prepared with the compounds of *S. khuzestanica*, *Z. multiflora*, and *O. volgarum* extractions, Honey, and Polyurethane with the equal ratio [24].

### MIC assay

The inhibitory effect of the ointment and its base is determined by disc diffusion method and then compared with the reference antibiotic [25].

The following steps were taken to determine the MIC:

1. Twenty-four-hour culture of *Pseudomonas aeruginosa PAO1* was prepared in Pseudomonas agar medium.
2. The stock of 0.5 McFarland (according to CLSI 2016) was prepared as a control in a separate test tube for opacity comparisons.
3. Colonies from the 24-h culture of *Pseudomonas aeruginosa PAO1* was inoculated into the physiological serum.
4. In addition to the 0.5 McFarland protocol, the method of measuring the OD in the tubes at 400 nm wavelengths and number of bacteria calculated based on CFU. Absorption of these samples with a spectrophotometer, in a wavelength of 625 nm, would be 0.08–0.13 that contains about 1.5 × 10^8^ CFU.
5. In seven tubes, 2 ml of MHB broth medium, 10 μl of *Pseudomonas aeruginosa* and the appropriate concentrations of the original stokes of ZOUSH ointment (extracts of the plant single, binary mixtures, triple mixture and ZOUSH ointment compounds) was inoculated. The proper concentration of primary stock: 5–10–20-40-80-160-320).
6. The tubes were incubated for 24–18 h in a 37 °C.
7. The incubation tubes were cultured on an MHA agar medium and then incubated at 37 °C for 18–24 h.
8. At the end of the incubation, time MIC was determined.MIC was calculated for each extract single, binary, a triple mixture, as well as a primary stock (ZOUSH ointment compounds).

### Determination of LD50

The LD_50_ value is determined to calculate the number of bacteria should be inoculated to the burned mouse, and after 5 min the bacteria are inoculated on the back surface of the mouse [26].

### Grouping of mice

The mice were classified into 5 groups. These groups and various treatments had shown in Table 1. Positive and negative control groups were used in this study. Ointment base (Oserin) as the treatment in the negative control group and Akbar 1 ointment (Iranian herbal ointment) and 1% Silver sulfadiazine as the treatment in the positive control group was used [27, 28].

**Table 1:** Grouping of mice in this study

Groups	Burn Wounds	Exposure to bacteria	No. mice	Ointment
Group 1 (negative control)	✓	✓	20	No Ointment
Group 2	✓	✓	20	Base Ointment (without active ingredient)
Group 3	✓	✓	20	ZOUSH Ointment
Group 4 (positive control)	✓	✓	20	Akbar 1 Ointment (herbal ointment)
Group 5 (positive control)	✓	✓	20	Silver sulfadiazine Ointment

### Burned mouse model

Burns are induced on the back surface of the BALB/c mice according to Ian Alan Holder method [29].

The mice used in this study were female BALB /c mouse old 6–9 weeks. In order to make the second degree of burns, Ian Alan Holder method as following steps carried out.

1. First, the mice were anesthetized by intraperitoneal injection of xylazine-ketamine anesthetics. 9.5 cc ketamine was mixed with 0.5 cc xylazine and is used as stock of anesthetics. 0.1 cc was injected for every mouse.
2. Dorsal surface hair was removed using a cream in a dimension of 3 × 3 cm^2^.
3. The back of the body was disinfected with 70% alcohol.
4. Aluminum pieces were pre-designed in 2 × 2 cm^2^ in size, and then sprayed with 96% alcohol, and then the flame was placed on the back surface of the mouse-free hair. Thus, 30% of the skin of the animal suffered from grade 2 burns.
5. To prevent dehydration of the animal, 0.5 ml of normal saline was injected subcutaneously into the animal’s back.
6. Five minutes after burning the animal was injected with 20 μl suspension with a sub-lethal half-Mcfarland concentration at the site of the wound and then spread.

### Treatment

The treatment period of the mouse was started 24 h after burning. Treatment of each group was performed according to the schedule and was performed as two times in the morning and afternoon at certain times. Also, the amount of ointment used was the same for each mouse.

The active ingredients of ZOUSH Natural Ointment include *S. khuzestanica*, *Z. multiflora* and *O. volgarum*, Honey and Polyurethane. The active ingredient natural Akbar 1 Ointment was Onosma dichroanthum Boiss, *Ricinus communis*, the essential oil of Mentha and *Z. multiflora*. Also, the active ingredient is silver sulfadiazine, silversmith (10%) and synthetic sulfadiazine (1%) of the sulfonamide group [30, 31].

### Bacterial count

The specimens were prepared from each group, in fifth , tenth , and fifteenth and twentieth days after treatment, the number of mice was selected for microbiological, Pathology and biochemical studies. The following operations were performed for each mouse:

1. As much as a standard loop, blood was cultured from the animal’s heart on a medium of Blood agar.
2. At the end of the phase, the rat liver was removed and inserted into microtubes.
3. The liver was weighed and its average was about 1 g per mouse.
4. In the next step, 100 μl serum sterilized physiology with supplements was added to the microtube and pipetted.
5. Then, 10 μl of microtube solution was cultured on Blood Agar.
6. The cultured plates (blood and liver) were incubated for 24 h at 37 °C. [32, 33].

### Wound culturing

First sampling was done with sterile swabs from the wounds. Then, the swab was placed inside sterile tubes containing 0.5 ml of physiological saline then culture carried out on Blood Agar. The cultured samples were incubated for 18–24 h at 37 °C. Then, the colonies examined by morphological and biochemical tests to determine the identity of isolated strains [34, 35].

### Real-time RT PCR

#### Gene silencing *exoS*, *lasA,* and *gyrA*

The inhibitory effects of ZOUSH ointment with MIC concentration on *lasA* and *exoS* genes and housekeeping gene gyrA were investigated using Real-time RT PCR technique. [25, 36, 37]

The inhibitory effect of ZOUSH ointment with MIC concentration on the internal housekeeping DNA Gyrase A is investigated using Real-time RT PCR. [25]

### Statistical analysis

Statistical analyses were carried out by descriptive and statistical methods (SPSS 24).

## Results

### MIC assay results

#### Measurement of MIC values for ZOUSH ointment

Table 2 shows the results of MIC values for *P. aeruginosa PAO1* using broth microdilution and broth microdilution techniques according to CLSI 2016.

**Table 2:** MIC Results for ZOUSH Ointment compounds: active ingredients as single, dual, triple

Substances	MIC
*Satureja khuzestaniea*	0.079 $\frac{\mu g}{ml}$
*Zataria Multiflora*	0.079 $\frac{\mu g}{ml}$
*Mentha Mozaffariani Jamzad*	0.158 $\frac{\mu g}{ml}$
Honey	0.079 $\frac{\mu g}{ml}$
*Satureja khuzestaniea*, *Zataria multiflora*	0.059 $\frac{\mu g}{ml}$
*Satureja khuzestaniea*, *Mentha Mozaffariani Jamzad*	0.079 $\frac{\mu g}{ml}$
*Zataria multiflora*, *Mentha Mozaffariani Jamzad*	0.079 $\frac{\mu g}{ml}$
*Satureja khuzestaniea*, *Zataria multiflora*, *Mentha Mozaffariani Jamzad*, Honey, Polyurethane	0.039 $\frac{\mu g}{ml}$

The results showed that the ZOUSH ointment compounds had better and greater antibacterial effects compared to any of the substances as single, dual mix and triple mix. On the other hand, since the extractions were color combinations, so all tubes were cultured on Muller Hinton agar to determine the MIC value using broth microdilution and broth microdilution techniques; the first non-growth plate was considered as MIC.

The results of the table demonstrated that the MIC value of the ZOUSH ointment mixture has decreased, indicating better and greater antibacterial effects of the ZOUSH ointment combinations compared to any of the substance as single, dual mix and triple mix. Result mean in per reaction was calculated. Results analyzed with SPSS version 24 with *p* values < 0.05 that indicated to be meaningful.

### Disc diffusion results

#### Antibiogram

To determine the resistance and sensitivity of *P. aeruginosa PAO1*, the antibiogram test was performed for Gentamycin (10 μg), Imipenem (10 μg), Meropenem(10 μg), Polymyxin B (300 u) and Tobramycin(10 μg) according to CLSI 2016 by Kirby-Bauer method that P. aeuginosa was sensitive to all antibiotics.

### Disc diffusion for ZOUSH ointment compounds

In order to determine the sensitivity of *P. aeruginosa* PAO1, the antibiogram test was performed using the discs contain mixed extracts as single, dual and triple mix as well as primary seed stock mix (S. khuzestaniea, *Z. multiflora*, M. Mozaffariani Jamzad, and Honey) with different concentrations. The results indicated Disc diffusion with mixed compounds with Honey, Mentha Mozaffariani Jamzad, Satureja khuzestaniea and Zataria multiflora with concentrations 10, 13, 17 and 20 μg were 12, 14, 15 and 18 mm respectively.

### In vivo results

#### Liver and blood culture results

Four mice were randomly excluded from each group at intervals of 5 days and on days 5, 10, 15 and 20 after treatment and were killed for microbiological, pathological and biochemical studies. The results of blood and liver cultures at different phases are reported as follows:

In the phase of burning according to Ian Alan Holder method, only *P. aeruginosa* was inoculated into the burn wound, but in addition to *P. aeruginosa*, *S. aureus* was isolated from blood and liver cultures of different mice groups.

*Staphylococcus* strains such as *S. aureus* are known as normal flora of the primates and mice body (201, 202). Therefore, isolated *S. aureus* was not counted.

### Counting *P. aeruginosa* isolated from liver cultures

The results of the mean count of *P. aeruginosa* isolated from the liver cultures in the exclusions 1 to 4 at intervals of 5 days were presented in the following 5 based on CFU/g. First exclusion of mice was done 5 days after treatment, second exclusion 10 days after treatment, third exclusion 15 days after treatment, and fourth exclusion (final) 20 days after treatment (Table 3).

**Table 3:** Mean count of *Pseudomonas aeruginosa* isolated in each study phase. (CFU/g)

Groups	Study 1	Study 2	Study 3	Study4
Base Ointment (without active ingredient)	906	2778	2222	1667
No Ointment	725	2223	1945	1111
Akbar 1 Ointment (herbal ointment)	817	277	139	95
Silver sulfadiazine Ointment	214	1850	184	18
ZOUSH Ointment	123	97	70	0

### Real-time PCR results

The results of ZOUSH ointment effect on the expression of *exoS*, *lasA*, *lasB* and housekeeping gene using Real-time RT-PCR technique have been shown in our study.

**Fig. 1:**
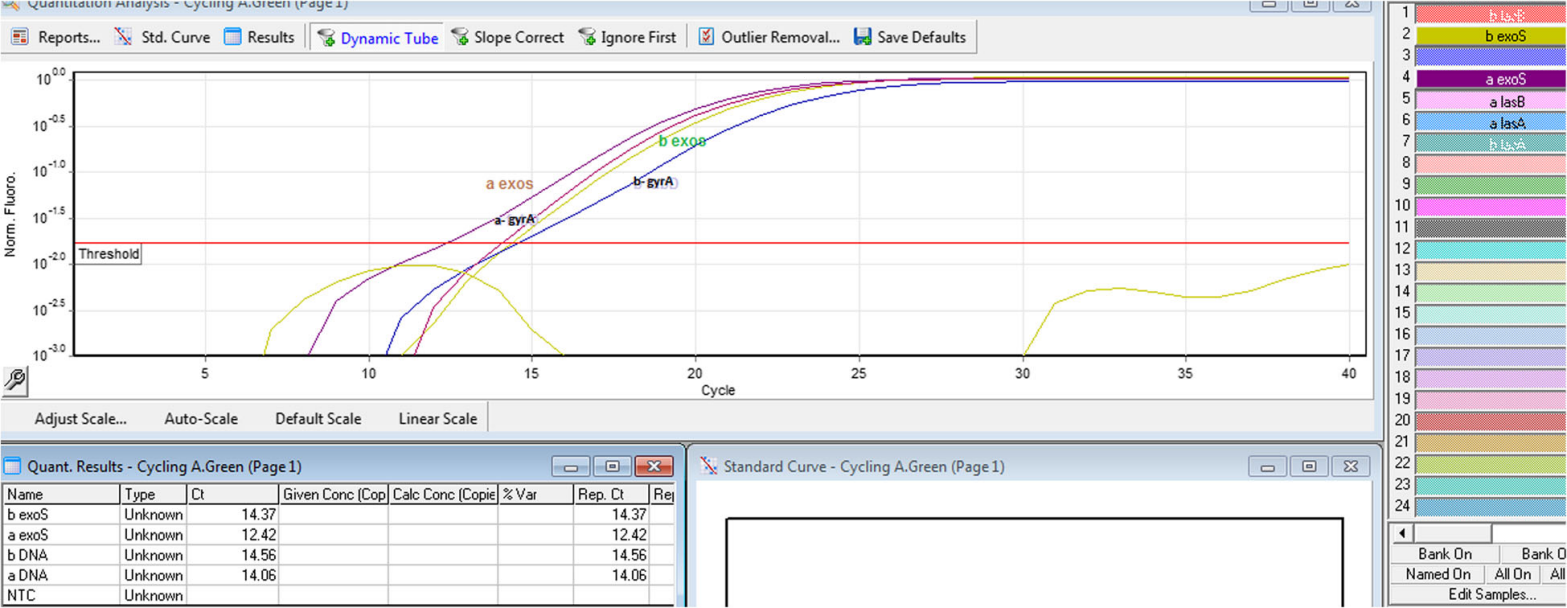
*exoS* gene amplification curve using Real-time RT-PCR technique: As outlined in the figure, the Ct level of the *exoS* gene was different before and after treatment (Ct before treatment = 14.32 and Ct after treatment = 12.42). However, there was no change in the Ct level of the *fabD* gene before and after treatment (Ct before treatment = 14.56 and Ct after treatment = 14.06). Therefore, the amount of the *exoS* gene expression changed after treatment, while the *FabD* gene expression had no change before and after treatment

**Fig. 2:**
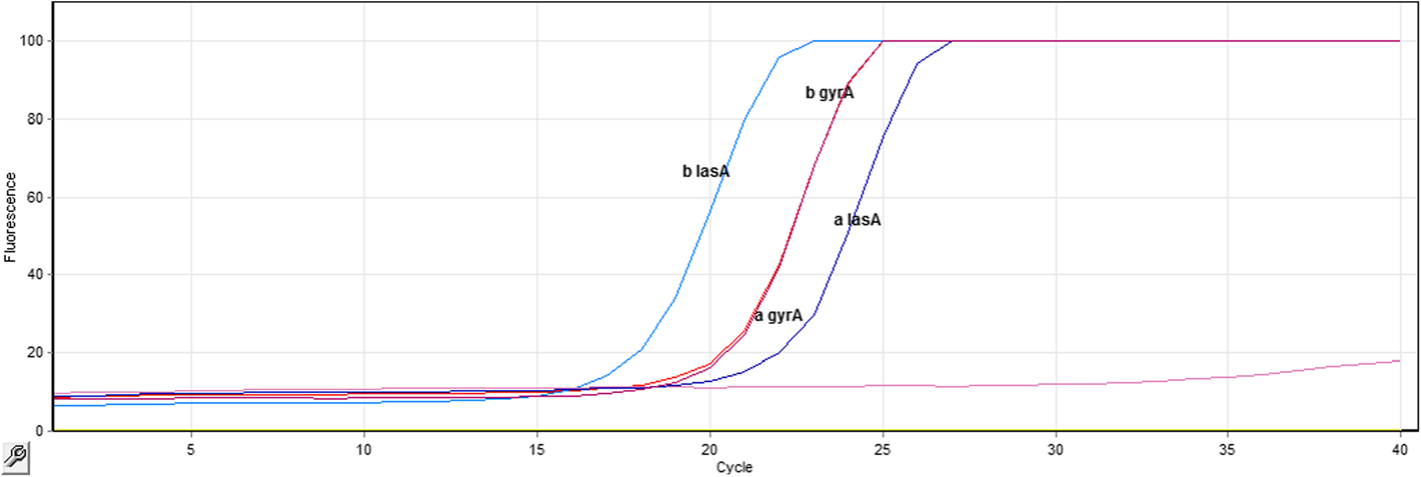
*lasA* gene amplification curve using Real-time RT-PCR technique: As outlined in the figure, the Ct level of the *lasA* gene was different before and after treatment (Ct before treatment = 12.64 and Ct after treatment = 10.38). However, there was no change in the Ct level of the *gyrA* gene before and after treatment (Ct before treatment = 14.56 and Ct after treatment = 14.06). Therefore, the amount of the *lasA* gene expression changed after treatment, while the *gyrA* gene expression had no change before and after treatment

**Fig. 3:**
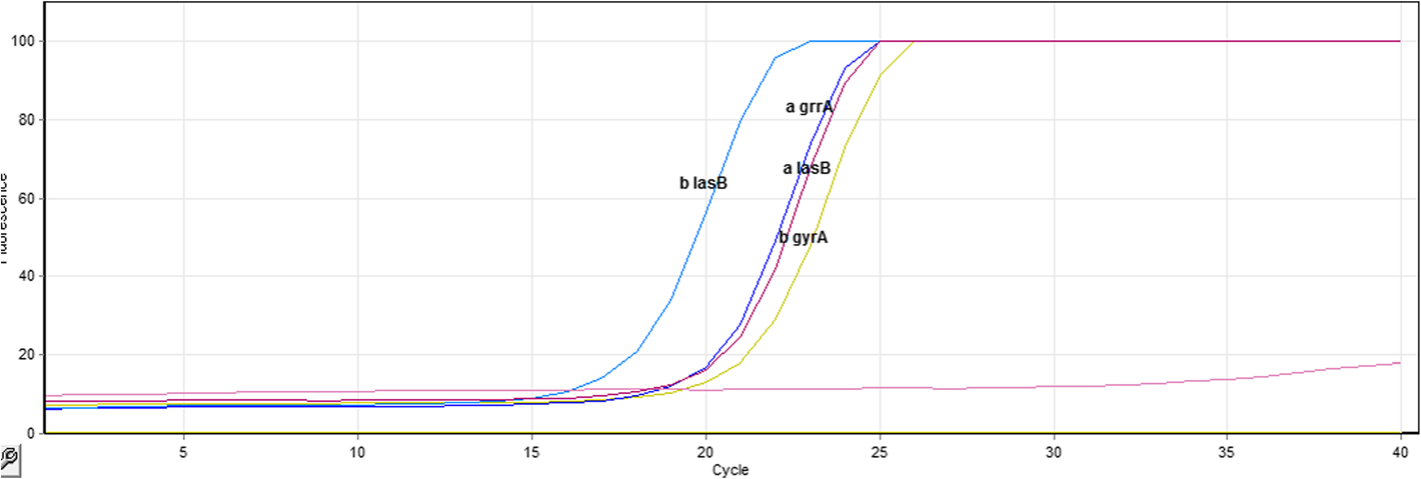
*lasB* gene amplification curve using Real-time RT-PCR technique: As outlined in the figure, the Ct level of the *lasB* gene was different before and after treatment (Ct before treatment = 13.24 and Ct after treatment = 12.64). However, there was no change in the Ct level of the *gyrA* gene before and after treatment (Ct before treatment = 14.56 and Ct after treatment = 14.06). Therefore, the amount of the *lasB* gene expression changed after treatment, while the *gyrA* gene expression had no change before and after treatment

The applied Biosystems device (Model Step One Plus) presents automatically all data on the Real-time RT-PCR reaction after each test and saves as an eds file. Each eds file contains the raw graph of the reaction progress in exponential mode. This file involves the test results as Amplification plot, Multicomponent plot, Melt curve, QC summery, Row data plot and Multiplot view. Amplification plot of Real-time RT-PCR reaction for *exoS*, *lasA*, *lasB* and *gyrA* genes was shown in Figs. 1, 2 and 3.

### The results of ZOUSH ointment effect on the *exoS* gene expression

For each sample, Real-time RT-PCR reaction was performed before and after treatment. Meanwhile, only one diagram of each reaction is given in below and no other reaction information is provided because of similarity (Fig. 1).

### The results of ZOUSH ointment effect on the *lasA* gene expression

For each sample, eight Real-time RT-PCR reactions were performed before and after treatment. Meanwhile, only one diagram of each reaction is given in below and no other reaction information is provided because of similarity (Fig. 2).

### The results of ZOUSH ointment effect on the *lasB* gene expression

For each sample, eight Real-time RT-PCR reactions were performed before and after treatment. Meanwhile, only one diagram of each reaction is given in below and no other reaction information is provided because of similarity (Fig. 3).

### Relative quantification results for real-time RT-PCR reactions

The relative quantification method measures relative changes in the expression levels of a gene mRNA relative to a housekeeping gene mRNA. In this method, unlike absolute quantification, there is no need to a standard curve, and the housekeeping genes that have stable expression are measured as reference genes.

The relative calculation of the gene expression according to the Livak method (ΔΔCt method), also known as _2_^-ΔΔCt^ or comparative Ct method, is performed without modifying its efficacy, and it is assumed that the desired doubling of target and reference DNA genes runs throughout each cycle.

Given the information, such as Ct, obtained from the amplification curve of the genes studied in the Real-Time RT-PCR method, the mean ΔCt before and after treatment was determined for each of the genes; subsequently, Cts were normalized. Figure 2 shows the mean Cts for each gene before and after treatment. This assay was shown for various genes using the following formula.The expression decrease virulence genes after treatment contain 2.73 fold for exoS gene, 3.38 fold for *lasA* gene and 1.07 fold for *the lasB* gene. The resulting analysis by SPSS version 22 demonstrated relation gene expression before and after treatment is meaningful (sig = 0).

## Discussion

*Pseudomonas aeruginosa* is an important opportunistic gram-negative bacterium that causes high mortality in immunocompromised patients. This bacterium is the third agent of hospital-acquired infection and the second pathogen of burn wounds [38].

Burn is one of the most serious medical conditions, which affects whole physical and mental aspects, and is capable of infecting people of all ages [39].

Prevention of *P. aeruginosa* spread in hospital settings is difficult due to the intrinsic and acquired resistance of this bacterium to many antibiotics. Despite many scientific advances in the treatment of burns, it remains one of the major public health problems around the world, especially in developing countries [40, 41].

Traditional medicine plays an important role in the treatment and prevention of these bacteria. The efficient method is needed for immediate treatment of this bacterium due to therapeutic dilemmas, drug resistance and high mortality due to *P. aeruginosa* [42].

Carvacrol is a major component of the *S. khuzestaniea* extract, which inhibits the ATPase activity and increases the bacterial cell membrane permeability, and hence enhances the membrane permeability of antibacterial agents [43].

Farzaneh et al. determined that carvacrol, γ-terpinene, and p-cymene constitute the major components of *S. khuzestaniea* [44].

Esmaeili et al. reported the inhibitory effect of *S. khuzestaniea* on *P. aeruginosa* [21].

Qing-huan Yin et al. examined the anti-inflammatory and apoptotic effects of carvacrol on hepatocellular carcinoma cell line HepG-2 and observed its anticancer effect [45].

Kamdem et al. showed that carvacrol had no specific effect on *Listeria monocytogenes* at 37 °C but was effective at 55 °C [46]. Mahbubi et al. studied the antibacterial properties of *S. khuzestaniea* essential oil on clinical isolates of *Escherichia coli* [47]. Jourabi et al. examined the antimicrobial effects of *S. khuzestaniea* and *Z. multiflora* with broth microdilution method on *E. coli*, *S. aureus*, *C. albicans* and *Aspergillus niger* [48]. In the study of Abbasi et al., the MIC of *S. khuzestaniea* was 80 μg/ml for *P. aeruginosa* [25].

In the study of Jalalvand et al., the MIC of *S. khuzestaniea* was reported 0.5 μg/ml for *P. aeruginosa* [49]; but in the present study, the MIC was 0.079 μg/ml for *S. khuzestaniea*.

In a study by Amiri et al., the antibacterial properties of *S. khuzestaniea* were examined on infectious bacteria in the respiratory tract [50].

In a study by Ghodrati et al., the antibacterial effect of *S. khuzestaniea* was performed on *C. albicans*, *E. coli*, *S. epidermidis* and *B. cereus* using disc diffusion and broth microdilution techniques [51].

In studies conducted by investigators, antibacterial effects these plants reported [21–39].

In the group treated with Akbar 1 ointment, the mean count of isolated *P. aeruginosa* was decreased, but this rate of decline was much lower than the rate of treatment with ZOUSH ointment. The mean count of isolated *P. aeruginosa* in treatment with Akbar 1 ointment after 20 days of treatment was equal to 10 days of treatment with ZOUSH ointment. These results indicate the bactericidal feature of the ZOUSH ointment had nearly two-fold higher effects.

In the group treated with silver sulfadiazine ointment, the mean count of isolated *P. aeruginosa* in the exclusion 2 had a significant increase compared to the exclusion 1, and two mice died in this group between the exclusions 1 and 2, due to the sudden increase in the mean count of the bacteria, and thus inability of the body. The silver sulfadiazine ointment was used to control the infection, which eventually occurred death. Moreover, the mean count of isolated *P. aeruginosa* in treatment with silver sulfadiazine ointment was decreased since the 10th day of the treatment. However, the bactericidal feature in the group treated with ZOUSH ointment was much higher, so that the mean count of isolated *P. aeruginosa* after 15 days of treatment with silver sulfadiazine ointment was even higher than the mean count of isolated *P. aeruginosa* after 5 days of treatment with ZOUSH ointment. These results indicated the bactericidal feature of ZOUSH ointment with an effect three times higher than to silver sulfadiazine ointment.

In the group treated with ZOUSH ointment, during the treatment, the mean count of isolated *P. aeruginosa* not only was reduced but also was zero in 20 days treatment. However, this result was not achieved in the groups treated with Akbar 1 and silver sulfadiazine ointment. These results also indicated the higher bactericidal feature of ZOUSH ointment compared to Akbar 1 and silver sulfadiazine ointments.

In the group treated with the base ointment as well as the untreated group, the mean count of isolated *P. aeruginosa* 5 days after the treatment was higher than the mean count of isolated *P. aeruginosa* 10 days after the treatment. It can be due to the inability of the immune system to eliminate the bacterium. However, the mean count of isolated *P. aeruginosa* 10 days after the treatment was less than the mean count of isolated *P. aeruginosa* 15 and 20 days after the treatment, which could be due to the improvement of the immune system ability to control the infection.

### Burn ointment test

In a study by Hua-Liang Li et al., the effect of Crocodile oil burn ointment (COBO) was evaluated on Wistar rats with second-degree burns. The analgesic and anti-inflammatory effects of COBO ointment were studied [52]. In survey ZOUSH ointment duration of treatment was 20 days, silver sulfadiazine ointment was used as a positive control.

In a study by Pannerselvam et al., the effect of silver nanoparticle-impregnated cotton fabric was investigated on the healing rate of wound burns in Wistar rats versus commercial ointments-impregnated cotton fabrics. The results indicated a faster improvement in the group treated with silver nanoparticles-impregnated with cotton fabric compared to other groups. The treatment period was 18 days, which is close to the duration of treatment with ZOUSH ointment [53]. Kittana et al. showed that *Ephedra alata* aqueous extract has the ability to heal wounds in animal models and investigated the effect of the *E. alata* aqueous extract ointment with polyethylene glycol ointment base versus placebo ointment. Phytochemical studies showed that *E. alata* aqueous extract contains flavonoids, alkaloids, phytosterols, phenolic compounds, volatile oils, and tannins. The *E. alata* aqueous extract ointment had the good effect on wound healing compared to placebo ointment. In this study the effects of ZOUSH ointment, carvacrol and thymol were the active ingredients of *S. khuzestaniea*, *Z. multiflora*, and *O. vulgar*, and the results showed wound healing in compared with the non-treated group and the group treated with ointment base [54].

To evaluation the effect of ZOUSH ointment, the antibacterial properties of ointment compositions was measured against *P. aeruginosa* PAO1, the inhibition zone diameter for ZOUSH ointment was reported 188 mm using the 60 μg/ml disk.

### Study of gene expression

Gene expression is a process resulting in the synthesis of a functional product using information stored in the gene. The gene expression is evaluated by several methods, such as Real-time PCR [55–59].

In this study, Real-time RT-PCR technique was used to study the effect of ZOUSH ointment compounds on inhibition the expression of *exoS*, *lasA*, *lasB* and *gyrA* genes in *P. aeruginosa*. Most of the studies have been conducted so far to investigate the antibacterial properties herbal compounds used MIC, disk diffusion, and RT-PCR methods.

In a study by Jalalvand et al., the effect of *S. khuzestaniea* was investigated on the *mexR* and *mexA* genes in *P. aeruginosa*. The results showed that low expression of these genes was due to carvacrol compounds [49]. In a research conducted by Moradi et al., the results indicated the effect of *S. khuzestaniea* on reduction the expression of virulence genes *P. aeruginosa* [60].

In a study by Esmaeili et al., the inhibitory effect of *S. khuzestaniea* on the expression of exoenzyme S, exotoxin A, secretory systems and efflux pump genes of *P. aeruginosa* was investigated by semi-quantitative RT-PCR technique [61].

Abbasi et al. identified the inhibitory effect of *S. khuzestaniea* on exotoxin A *P. aeruginosa* by semi-quantitative RT-PCR technique [25].

Jalali et al. employed the semi-quantitative RT-PCR technique to optimize the analysis of CPTI gene expression in trout fish liver [62].

Saghi et al. investigated the effect of *S. khuzestaniea* and *Z. multiflora* essential oils on the control of aphA-6 gene expression in MDR *Acinetobacter baumannii* by Real Time-PCR technique. The results showed that carvacrol is the main compound of *S. khuzestaniea* and thymol is the major compound of *Z. multiflora*. The MIC was 0.3 μl/ml for *S. khuzestaniea* and 0.45 μl/ml for *Z. multiflora*. The aphA-6 gene encodes aminoglycoside transferase enzyme, thereby providing resistance [63].

Bahador et al. studied the effect of *S. khuzestaniea* alcoholic essential oil on the expression of the gene associated with *Acinetobacter baumannii* biofilm by the Real-Time PCR technique. The results showed that the presence of 90.88% carvacrol in the essential oil of *S. khuzestaniea*, and MIC was reported 0.3 μg/ml for *S. khuzestaniea* essential oil. In this study, the results of Real-time PCR were analyzed by SPSS software and ANOVA test. [25].

The present study was carried out to investigate the effect of herbal ZOUSH ointment on inhibition the expression of *exoS*, *lasA*, *lasB* and *gyrA* genes in *P. aeruginosa* by Real-time RT-PCR technique.

## Conclusion

We can conclude that in a burn wound model in mice, ZOUSH ointment was able to decrease the healing process both biochemically and statistically as compared to SSD and the control group. Through its evident ant virulence gene effects, ZOUSH can be used as an alternative agent to wound healing therapies in the future. However, wound tissue healing is a multi-factorial process involving the groups of enzymes, receptors, gene silencing and healing. Therefore, further research is needed for the elucidation of the role of these processes on the rabbit and human model for the observed action of ZOUSH.

## Abbreviations

CT: Cycle threshold
MDR: Multiple drug resistance
MIC: Minimum inhibitory concentration
SSD: Silver sulfadiazine
XDR: Extensively drug resistance
ZOUSH ointment: It is trade name of our natural ointment
